# Struggling to Understand the NEC Spectrum—Could the Integration of Metabolomics, Clinical-Laboratory Data, and Other Emerging Technologies Help Diagnosis?

**DOI:** 10.3390/metabo14100521

**Published:** 2024-09-26

**Authors:** Kosmas Sarafidis, Eleni Agakidou, Angeliki Kontou, Charalampos Agakidis, Josef Neu

**Affiliations:** 1 1st Department of Neonatology, School of Medicine, Aristotle University of Thessaloniki, 54642 Thessaloniki, Greece; eagakidou@auth.gr (E.A.); angiekon2001@yahoo.gr (A.K.); 2 1st Department of Pediatrics, School of Medicine, Aristotle University of Thessaloniki, 54642 Thessaloniki, Greece; cagakidis@auth.gr; 3 Department of Pediatrics, Division of Neonatology, University of Florida, Gainesville, FL 32611, USA; neuj@peds.ufl.edu

**Keywords:** neonate, intestinal injury, definition, subtyping, omics

## Abstract

Necrotizing enterocolitis (NEC) is the most prevalent and potentially fatal intestinal injury mainly affecting premature infants, with significant long-term consequences for those who survive. This review explores the scale of the problem, highlighting advancements in epidemiology, the understanding of pathophysiology, and improvements in the prediction and diagnosis of this complex, multifactorial, and multifaced disease. Additionally, we focus on the potential role of metabolomics in distinguishing NEC from other conditions, which could allow for an earlier and more accurate classification of intestinal injuries in infants. By integrating metabolomic data with other diagnostic approaches, it is hoped to enhance our ability to predict outcomes and tailor treatments, ultimately improving care for affected infants.

## 1. Introduction and Methodology

What is termed “necrotizing enterocolitis” (NEC) is the most common and potentially fatal diagnosis related to intestinal injuries in premature infants, with serious short- and long-term consequences for survivors. It has become clear that a paradigm shift is needed if we are to make progress in the prevention and management of the various conditions placed under the umbrella of NEC. 

In their 1986 key review on NEC and its treatment based on staging criteria, Walsh and Kliegman discussed the future goals for managing this condition as follows: “It is obvious that we are far from a consensus on the etiology or etiologies of NEC. It is likely that NEC may be more than one disease process. Future studies must clearly differentiate patients with different severities of illness, perhaps by using staging criteria and classification. We can then begin to investigate and define the pathogenesis of NEC. Once the pathology is understood, we can identify those infants at risk and possibly prevent NEC” [1]. Unfortunately, not much progress has been made, largely because of the heterogeneity of what is being called “NEC” and the lack of a definition. 

In this narrative review, we explore the scale of the problem, the advancements made in epidemiology, and the understanding of pathophysiology, prediction, diagnosis, and outcomes of NEC. Additionally, we focus on the role of metabolomics in achieving these goals. It is to be noted as a disclaimer to the following review that when we discuss “NEC”, there is currently no single accepted definition, and with newly developing technologies such as artificial intelligence and integrated multiomics, it is likely that the paradigm will shift to newly defined clusters of intestinal injury and the term “NEC” will become an anachronism. 

The literature review method included searching electronic databases (PubMed, Scopus, and the Cochrane Library) for articles on NEC, neonatal intestinal injury, and metabolomics published from January 2010 up to May 2024. Moreover, a manual search of the reference lists of the included studies was conducted to find additional relevant articles. The MeSH terms used included “necrotizing enterocolitis”, “spontaneous intestinal perforation”, “gut injury”, “intestinal injury”, “near-infrared spectroscopy”, “microbiome”, “metabolome” “metabolomics”, “omics”, “biomarkers”, “diagnosis”, “ultrasound”, “radiology”, “neonatal care”, “neonatal sepsis”, “epidemiology”, “classification”, “outcome”, “artificial intelligence”, “machine learning”, “definition”, “diagnosis”, “laboratory investigation”, “preterm infants”, “term infants” “randomized controlled trials”, “review”, and “systematic review. The search included clinical studies (randomized clinical trials [RCTs], cohort studies, and case-control studies), case reports, and any kind of review studies. We included studies published as full publications in English.

## 2. The Current Problem of NEC 

### 2.1. Epidemiology

The incidence of NEC is inversely related to gestational age (GA) and birth weight (BW) [2]. Previous studies reflecting advancements in perinatal medicine-neonatology, including surfactant administration, have shown a reduction in the incidence of NEC despite the increased survival of extremely premature infants [3]. The implementation of comprehensive NEC prevention initiatives such as breast milk feeding, standardized feeding protocols, transfusion guidelines, and antibiotic stewardship has been associated with the decline in the diagnosis [4]. In contrast, other investigators have reported an increase in the incidence of NEC over time, attributing it to improvements in neonatal care and the better survival of premature infants, as well as enhancements in diagnosis and reporting [5,6].

In a prospective registry involving extremely low birth weight (ELBW) infants born in the United States (1993–2012), where survival was found to most markedly increase in infants born at 23- and 24-weeks’ gestation, stage 2–3 NEC was diagnosed in 7%, 13%, and 9% of the studied population in the years 1993, 2008, and 2012, respectively. Still, a wide variation in the incidence of NEC was reported among extremely preterm neonates in American hospitals [7]. Data from the Spanish Neonatal Network involving very low birth weight (VLBW) infants showed a stable incidence of NEC (8.8%) over the study period (2005 to 2017) despite the improvement regarding protective measures (exclusive breast milk, probiotics) [8]. A relatively recent systematic review of the incidence of NEC in high-income countries revealed considerable variation in the NEC rate. It ranges from 2% to 7% in preterm infants with a GA less than 32 weeks and from 5% to 22% in those with BW less than 1000 g [9]. Globally, seven out of every 100 VLBW infants receiving neonatal intensive care are likely to develop NEC [5].

Although NEC is predominantly a condition associated with prematurity, it can also occur in near-term neonates and term infants admitted to a Neonatal Intensive Care Unit (NICU) for other reasons. Full-term infants were reported to account for approximately 10% of NEC cases [10]. Nevertheless, NEC in more mature neonates usually occurs in the first week after birth [11], whereas in very premature neonates it typically appears around the fourth week after birth [3]. A study from the Canadian Neonatal Network found that among infants born before 33 weeks of gestation, NEC tends to present at a mean age of 7 days in more mature infants, while the onset of NEC is delayed to 32 days of age in smaller, lower GA infants [12].

### 2.2. Outcome of Infants with NEC 

NEC mortality has been reported as decreasing [8,13] or stable [6] over the years. Of the premature infants with NEC, approximately one-third to one-half will require surgery. It is precisely this subgroup of subjects that exhibits higher mortality rates, ranging up to 20–30% [8,11]. In ELBW infants with surgical NEC, mortality rates were found to be as high as 50.9% [14]. 

On the other hand, in addition to death, several common neonatal mortalities (bronchopulmonary dysplasia (BPD), late-onset sepsis (LOS), cystic leukomalacia, retinopathy of prematurity, acute kidney injury) have been associated with the inflammation putatively caused by NEC [8]. A recent systematic review and meta-analysis revealed that preterm infants with NEC have a higher risk of neonatal brain injury and neurodevelopmental disorders compared to non-NEC infants, especially those treated surgically. Furthermore, NEC is associated with delays in motor, cognitive, and language development, as well as attention deficits in children [15]. Lastly, NEC is the leading cause of short bowel syndrome in neonates [16], necessitating a multidisciplinary approach to improve long-term survival and enteral autonomy [17]. The international acceptance and use of common core long-term outcomes in NEC trials is hoped to improve scientific knowledge on the consequences of the NEC from the neonatal period to adulthood [18].

### 2.3. Pathophysiology 

Despite many years of systematic scientific research, NEC largely remains a scientific mystery. It is remarkable that even today, the nature of the disease and its pathophysiological mechanisms have not been fully elucidated. Nevertheless, the prevailing opinion is that NEC represents a complex set of entities that can arise from various pathological conditions. There is increasing acceptance that “NEC” may simply serve as an umbrella term for a spectrum of multiple conditions with different pathophysiologies, some of which lead to intestinal necrosis [19,20]. Moreover, different predisposing factors have been recognized in premature and term newborns. A brief description of current concepts of NEC pathophysiology follows. Readers may find more extensive and updated reviews on pathogenesis elsewhere [11,21,22]. 

In premature infants, gut immaturity, enteral feeding, microbial dysbiosis, local ischemia, and/or reperfusion injury, as well as inflammation, are considered to play significant roles in the development of NEC [23]. The importance of intestinal immaturity is indicated by the strong, inverse relationship between GA and NEC incidence and associated mortality [4,24]. The gastrointestinal tract of preterm infants is considered to be in a hyperreactive state, exhibiting excessive inflammation [25]. Additionally, their intestine is colonized with pathogenic microbes (dysbiosis) [26], and various clinical conditions (thus the term multifactorial) may lead to gut ischemia [27]. Toll-like receptor 4 (TLR4), a receptor that identifies lipopolysaccharides from Gram-negative bacteria, appears to have a significant impact on NEC development by triggering intestinal injury via TLR4 receptor signaling and impairing mucosal repair in premature infants [28,29]. Breast milk is protective against the onset of NEC by suppressing TLR4 in the intestinal epithelium through the activation of the epidermal growth factor receptor [30]. In this context, TLR4 inhibitory molecules hold promise as novel preventive or therapeutic approaches in NEC. However, their role and value require further evaluation in future studies [29].

Intestinal microbiome is another crucial regulatory factor in intestinal immune defense. The presence of commensal bacteria helps protect against bacterial translocation and the exacerbation of local inflammation. Conversely, dysbiosis, or alterations in the intestinal microbiota, is increasingly recognized as having a dominant role in NEC [21,31]. Moreover, decreased Firmicutes and Bacteroidetes, along with increased Proteobacteria, have been documented to precede the onset of NEC [32,33]. The differences in taxonomic profiles have been associated with common clinical variables such as antibiotic exposure, formula feeding, and mode of delivery [26]. Interestingly, emerging evidence suggests that maternal prenatal stress and stress-induced microbial dysbiosis can predispose an infant to NEC by disrupting the microbial communities of both mother and child. It is possible that stress hormones directly affect microbial proliferation influencing composition and diversity in the gut [34]. A recent systematic review by Moschino et al. on the metabolome and gut microbiota for predicting NEC and spontaneous intestinal perforation (SIP) summarizes data from 27 studies comparing microbiomics in neonates with and without NEC. It found that 11 out of 27 studies reported decreased bacterial diversity in NEC infants compared to those without NEC, while 12 studies did not find significant differences, and four studies lacked this information. This variability in gut microbiota composition among NEC infants may be due to differences in sampling conditions, measurement techniques, and software used. No specific bacterial species have been conclusively linked to NEC [35]. 

In term and late preterm infants, risk factors include hypoxemia-ischemia following perinatal asphyxia [36] or congenital heart diseases [37], as well as intestinal anomalies (e.g., aganglionosis or atresias) [11]. Feeding with formula has been attributed to an increased risk of NEC when compared to a mother’s milk feeding, but the evidence for causality from formula does not exist. The difference in risk between formula and mother’s milk is likely secondary to the protective bioactive components that have not been able to be replicated in formulas [38,39].

The potential genetic predisposition to NEC is also noteworthy. Initially suggested by epidemiological data among different ethnic groups and in concordant twins, the role of genetics in NEC has gained attention. Many promising genes associated with an increased or decreased risk of NEC have been identified because of advances in genetic research. Moreover, methylation-related differential genes, i.e., epigenetic changes, were identified and verified on genes primarily expressed on intestinal epithelial villus cells [40,41]. However, most of these studies have small sample sizes, involve infants with different NEC stages and ethnicities (based on the country of origin), and have not been validated in cohort or functional studies [42]. Furthermore, the heterogeneity of different forms of intestinal injury placed under the umbrella of NEC dilutes the subsets that may have a genetic origin. Future research utilizing novel genome-analyzing techniques on better-defined clusters of patients with intestinal injuries is expected to provide further insights into the genetic predisposition to NEC, particularly by incorporating other advanced technologies such as artificial intelligence and -omics (see below).

## 3. Definition and Staging 

There is no consensus on the exact definition of NEC [4]. The disease was first reported in the 1940s. However, a case described by Charles Billard in Paris in 1828, displaying clinical findings similar to those seen in NEC today, may represent the first report of the disease. Clinical and patho-anatomic characterization was achieved by Schmidt and Quaiser in 1952 [43,44]. In 1978, Dr. Martin Bell proposed a staging system that was used to assess which patients should undergo surgery [45]. Although the nature of NEC was extensively debated, the Bell staging system became increasingly used to describe and diagnose NEC. Up until the end of the 1980s, the Bell criteria remained the dominant method of NEC staging. 

Modifications were subsequently made (1986) by Walsh and Kliegman, resulting in the so-called modified Bell’s criteria. Specifically, each stage (I–III) was divided into two subcategories, including signs that differentiate between milder and more severe courses of the disease, such as absent bowel sounds, abdominal tenderness, and ascites, as well as laboratory parameters indicative of acidosis, thrombocytopenia, neutropenia, and disseminated intravascular coagulation. In stage I, symptoms from the gut are present, and NEC is classified as suspected since there are no pathognomonic signs. In stage II, definite NEC is diagnosed when intestinal pneumatosis is found on the abdominal X-ray. Stage III represents the advanced form of the disease, characterized by significant clinical burden and additional intestinal rupture (pneumoperitoneum) [1]. Pneumatosis intestinalis (air in the bowel wall), portal venous gas, and pneumoperitoneum are the most important findings on plain abdominal X-rays to confirm the diagnosis of NEC [46]. Nevertheless, in clinical practice, disagreement often occurs over signs on X-rays when it is unclear if findings are suggestive of pneumatosis or not [47]. More generally, there seems to be high interobserver and intraobserver variability in the interpretation of abdominal radiographs in infants with suspected NEC [48]. It is important to underline the fact that these staging criteria do not define NEC. 

Over time, it became evident that NEC is neither a uniform nor a well-defined disease entity, which calls into question Bell’s criteria [11,49]. Moreover, Dr. Bell himself acknowledged that SIP is distinct from NEC, and that the classification he developed never meant to include this disease [50]. There are several other reasons against the use of Bell’s criteria nowadays: (a) developed in the late 1970s to grade the degree of injury in premature babies, the original criteria now face limitations due to dramatic increases in the survival of ELBW infants. Due to their concomitant gut immaturity, these infants often present with symptoms from the gut (e.g., feeding intolerance and/or abdominal distension that are often during non-invasive ventilation) that can lead to misclassification as stage I NEC (lack of specificity), (b) there are diagnostic limitations; the criteria rely heavily on clinical and radiographic findings, which can be subjective and may not always correlate with the severity of the disease or the underlying pathological processes, (c) advanced diagnostic tools, such as ultrasound and near-infrared spectroscopy (NIRS), and biomarkers that possibly provide more precise and earlier detection of NEC are not incorporated into the criteria, and (d) once infants are diagnosed with definitive intestinal necrosis, the specific etiology remains multifactorial and pathologic specimens likely will not be able to provide mechanistic etiologic information. Several definitions of NEC have been proposed by organizations and academic centers, possibly being more effective in identifying NEC [51,52]; however, this fact simply highlights our inability to precisely define NEC.

## 4. Laboratory Investigation

### 4.1. Conventional and Promising Biomarkers

Due to the relative nonspecificity of current clinical and radiographic tests, various biomarkers have been evaluated for the assessment of NEC and its progression. Currently used biomarkers can be classified into hematological indices (such as total white blood cell count, absolute neutrophil count, immature to total white blood cell ratio, and platelet count) as well as acute phase reactants and immunological markers including acute phase proteins (such as C-reactive protein, procalcitonin, serum amyloid-A [SAA], platelet-activating factor, and hepcidin), toll-like receptors, cytokines (such as interleukins-6 and-8, tumor necrosis factor-α), as well as other chemokines, adhesion molecules, and growth factors. However, these biomarkers are nonspecific for NEC, and their diagnostic accuracy varies depending on NEC severity and the time-point during the course of NEC [53]. According to an international survey on the management of NEC, pediatric surgeons most frequently use platelets (99%), C-reactive protein (90%), and/or white cell count (83%) for the evaluation of NEC. Additionally, smaller proportions utilize lactate levels (43%) for assessment [54]. 

Organ-specific biomarkers indicating enterocyte injury and intestinal barrier dysfunction have also been evaluated for the prediction and diagnosis of NEC. Fecal calprotectin, intestinal fatty acid binding protein (i-FABP), claudin-3, and trefoil factor 3 (TFF-3) are among the most promising biomarkers for NEC diagnosis [53,55]. Current evidence suggests that fecal calprotectin levels are elevated in newborns suffering from NEC. Reported sensitivity ranges from 76% to 100%, while specificity ranges from 39% to 96.4% [56]. Plasma i-FABP may exhibit high specificity in infants with NEC. However, its usefulness is limited by its moderate sensitivity [57]. Moreover, urinary i-FABP appears to have limited value in the diagnosis of NEC [57] but the combination of urinary i-FABP, TFF-3, and SAA was reported to predict pneumatosis intestinalis [58]. Despite these biomarkers appearing to offer specificity and sensitivity, it is clear that they are not predictive biomarkers since the evidence for this is lacking. 

### 4.2. Ultrasound Examination

Ultrasound examination (US) has emerged as an alternative to the current standard usage of radiography for NEC diagnosis in the NICU setting. Apart from limiting exposure to radiation, this approach offers other advantages. It is non-invasive and can be readily performed at the bedside without limitations on frequent use. Moreover, various indices of interest can be evaluated, including bowel wall thickness, echogenicity, the presence of pneumatosis intestinalis, the rate of bowel wall perfusion, and the ability to determine the nature and estimate the amount of intra-abdominal fluid [53,59]. It is worth noting that pneumatosis intestinalis and portal venous gas can both be detected earlier during abdominal US compared to plain radiography. Additionally, the sensitivity of pneumatosis intestinalis and portal venous gas for diagnosing NEC stage > II was reported as high as 90% using ultrasound examination [60]. Other studies have demonstrated that combining sonographic and radiographic imaging features can aid in predicting the outcome of patients with NEC [61]. In a relevant systematic review and meta-analysis on the role of bowel ultrasound in predicting surgical management of NEC, it was concluded that this imaging technique may be useful for the early identification of high-risk infants with NEC who may benefit from more aggressive treatment, including surgery [62]. Interestingly, as shown in a previous survey, 50% of the surgeons request an intestinal Doppler ultrasonography in addition to plain X-rays in the work-up of patients with suspected NEC [54]. 

Overall, a growing body of evidence suggests that the inclusion of bowel ultrasound in our diagnostic toolkit may be beneficial for the more precise evaluation of infants at high risk for NEC. This raises further questions about the continued reliance on Bell’s criteria as the standard of practice for defining, diagnosing, staging, and treating NEC. Even if a definition of NEC is not currently available, ultrasound can still detect evidence of intestinal injury, even without a specific label.

### 4.3. Near-Infrared Spectroscopy

Alternations in intestinal microcirculation play a significant contributing role in the development of NEC [63]. NIRS is a relatively novel technology that utilizes infrared light to monitor regional tissue oxygenation noninvasively and continuously. Additionally, NIRS serves as a surrogate indicator of tissue perfusion in various organs [64].

In this context, NIRS is increasingly being utilized in neonatal research for the early diagnosis of brain injury [65], but also for monitoring splanchnic oxygenation in preterm infants at risk for NEC [66]. Studies have documented impaired intestinal oxygenation before the onset of clinical NEC in preterm infants, enabling the prediction of NEC development, on average, 2 days prior to the onset of the disease [67]. In another study, complicated NEC (defined as Bell’s stage IIB or death due to NEC) could be differentiated from uncomplicated NEC by measuring cerebral and splanchnic oxygenation. However, NIRS monitoring did not prove useful for distinguishing between definite NEC (Bell’s stages II and III) and no NEC (Bell’s stage I at most) at the onset of clinical signs in preterm infants [68]. An inherent issue with evaluating splanchnic oxygenation using NIRS is that the measurement can be affected by factors such as abdominal distension and fecal content. Nevertheless, as concluded in a recent review article by Howarth et al., continuous splanchnic NIRS monitoring may improve the clinical outcomes of preterm infants by alerting clinicians to relative gut hypoxia, allowing for timely interventions [69]. 

Currently, there is no conclusive evidence regarding NIRS and its ability to predict NEC, highlighting the need for further research on the role of the non-invasive monitoring of gut injury. 

## 5. Differential Diagnosing and Subtyping 

Undoubtedly, our comprehension of the complex factors contributing to the development of NEC has advanced over the years. However, the pathophysiology of NEC remains incompletely understood, partly due to the heterogeneous nature of the disease. 

Several case series have documented infants exhibiting signs and symptoms overlapping those of NEC, elegantly categorized into NEC subsets by Gordon et al. [4]. NEC in late preterm and full-term infants often stems from different disease triggers and presents at different times. Even within the subgroup of very preterm infants, the clinical presentation of advanced NEC and intestinal perforation can closely resemble that of SIP. Advanced NEC is characterized by extensive intestinal diffuse inflammation and ischemic necrosis, whereas SIP typically manifests as an isolated perforation of less than 2 cm without diffuse bowel pathology [55]. SIP is now recognized as a distinct clinical entity from NEC, with specific clinical, radiologic, and intraoperative findings [70]. The diffuse inflammation and ischemia observed on histopathology in infants with NEC are absent in SIP. However, recent challenges in conclusively distinguishing between SIP and NEC have led to speculation that both conditions may lie on the same spectrum of intestinal injury [71].

Theoretical yet challenging questions arise regarding whether the various triggers of gut injury share a common or distinct pathophysiological pathway. Taxonomy of subsets of intestinal injury becomes crucial as it aims to capture and catalog the divergent stressors that either trigger or potentiate the onset of NEC-like pathologies. Currently, sub-setting is based on the presence of a triggering event (such as following transfusion of packed red blood cells or administration of cow’s milk), the timing of appearance (as seen in SIP), and radiological and hematological results, with less emphasis on surgical and pathological findings. However, even this widely used approach has limitations, given the moderate agreement between preoperative, intraoperative, and pathological diagnoses [72]. Apparently, further research is warranted to enhance diagnostic accuracy in distinguishing SIP from NEC. While categorizing NEC cases represents a significant advancement, a comprehensive understanding of the pathophysiological mechanisms remains paramount.

Subtype discovery has emerged as a major focus of medical research. Various subtypes of sepsis and acute respiratory distress syndrome (ARDS) in critically ill adults have been identified using clinical data [73,74] or gene expression profiling [75]. Furthermore, ARDS subphenotypes have shown differential responses to fluid management strategies [76]. Similarly, in neonatology, BPD, a form of chronic lung injury mainly affecting preterm infants, may be categorized into different phenotypes based on the underlying organ or tissue involvement. Parenchymal lung disease, airway disease, or pulmonary vascular disease contribute differently to the pathophysiology and clinical presentation of BPD [77]. Interestingly, researchers have proposed applying the BPD framework to NEC, considering significant developmental phenomena and postnatal exposures affecting gut maturation prior to NEC development. NEC is often perceived as an abrupt process due to its sudden onset and rapid progression once diagnosed but this does not seem to be the case [78].

## 6. Novel Technologies to Delineate NEC

Different disease processes must be delineated before developing diagnostic and predictive biomarkers for accurate and early recognition of distinct intestinal injuries (currently all called “NEC”) subsets to provide the most appropriate treatment. A multifaceted approach, involving new technologies like artificial intelligence (AI) and omics, is expected to help redefine the entire spectrum of intestinal injury in critically ill infants.

### 6.1. Artificial Intelligence 

The unique ability of computers to rapidly process large amounts of data is well known and easily understood. In contrast, AI requires more explanation, especially for readers unfamiliar with the subject. Essentially, AI aims to match or exceed human intelligence and capabilities by imitating cognitive functions. This involves discovering or inferring information that may not be explicitly stated, as well as reasoning.

Machine learning (ML) is a field of AI that uses existing data and complex statistical analyses to develop predictions or make decisions [79,80,81,82]. Unlike traditional programming, statistical algorithms in ML can learn from data, improving their accuracy in predictions and decision-making as they process more information. There are three major types of ML: supervised learning, unsupervised learning, and the hybrid approach. As the name implies, supervised learning involves more human oversight, with predefined outcomes of interest and recognized influences. In contrast, unsupervised learning uses unlabeled input data, allowing the ML model to identify patterns or structures within the data that may be impossible for humans to detect [83,84]. Deep learning (DL) is a subset or subfield of ML known for its advanced performance. DL aims to mimic the human brain by creating models based on artificial neural networks and algorithms. These models, comprised of nodes and statistical relationships between them, function similarly to the human brain and learn from extensive datasets. The term “deep” refers to the multiple layers within these neural networks.

In recent years, AI has not only become increasingly prevalent in everyday human life but has also been integrated into various fields of medicine, including cancer, neurology, and cardiology [85]. Studies employing AI methods have shown improved accuracy in disease prediction and the identification of risks, particularly in sub-populations where traditional methods exhibit poor predictive accuracy. ML has been noted to effectively assign ARDS subphenotypes using readily available clinical data, potentially enabling prognosis and personalized treatment [86]. ML demonstrates significant potential in enhancing sepsis diagnosis and prediction, among other diseases of interest in critical care settings [87]. 

Omics data inputs can improve both the performance of ML models and precision medicine [88]. However, integrating and analyzing demographic/clinical characteristics along with large omics datasets (genetic, proteomic, and metabolomic) poses a challenge that demands more complex models for effective data handling and interpretation [83,89]. Various software tools have been developed, and ongoing innovations are addressing the challenge of integrating and analyzing demographic/clinical characteristics with large omics datasets [84]. However, there’s the issue of interpretability; AI models may not provide explanations for their results or decisions, posing challenges for clinicians. Nonetheless, this holistic approach can greatly enhance our understanding of biological alterations associated with diseases, surpassing what the human mind can achieve alone, ultimately leading to the discovery of new biomarkers [90].

### 6.2. Artificial Intelligence and NEC 

AI holds promise in transforming the care of neonates with NEC and advancing research, although there are relatively few publications on AI related to NEC. Moreover, a significant number of the existing data are based on single-center studies and, therefore, they have limited generalizability [91]. 

In a recent (2023) state-of-the-art review, McElroy and Lueschow discussed basic concepts of AI, highlighted advantages and limitations in the field, and, most importantly, summarized existing studies (n = 18) that have applied AI to NEC. It is important to note that these studies utilized “NEC” as a discrete outcome and the shortcoming of this is obvious from our previous discussion regarding the lack of a definition. Of these, 7 were multicenter and 11 single-center studies. Supervised ML was applied in 16 studies, unsupervised in two, and DL in one study. The models developed were primarily aimed at determining or predicting risk factors for NEC, differentiating medical and/or surgical NEC from controls, predicting NEC and distinguishing it from non-NEC conditions like feeding intolerance or SIP, and predicting the need for surgical intervention or prognosis of NEC [83]. Other studies focused on objectively scoring NEC severity or identifying important features for model decision-making. All models were based on clinical features, with some incorporating novel urine biomarkers or data related to metabolomics, stool microbiome, metagenomic taxonomy, and radiomics [83]. 

More recent data indicate that AI technology can assist clinicians in the early detection of LOS and NEC in the NICU, potentially with clinical and socioeconomic benefits. Compared with historical diagnosis, the ML model applied allowed a median time gain of ≤10 h (IQR, 3.1–21.0 h in diagnosis [92]), apparently allowing the prompts and timely application of measures to treat these two fast evolving diseases. Another ML study analyzed the maternal, prenatal, and postnatal factors acquired within 7 days of birth, in 16,385 VLBW infants registered in the Korean Neonatal Network. Beyond the identification of demographic/clinical variables (low GA and BW, arterial hypotension requiring treatment), an ML model was proposed for the early after-birth identification of VLBW infants at high risk for surgical NEC [93].

Contemporary definitions of NEC have been critically evaluated using AI compared to traditional statistical techniques. Beyond assessing the accuracy of NEC definitions, supervised ML identified important diagnostic features, ML allowed the identification of features. Notably, pneumatosis, despite having the highest observed feature importance score for NEC definitions, ranked only fifth in importance out of nine features in the most critical feature definition [51]. 

Supervised ML has been applied to determine the most common differentiating factors between NEC and SIP prior to the development of the event substantiated at surgery [79]. Researchers also utilized unsupervised ML to re-evaluate the classification of neonatal intestinal diseases. In a retrospective study, infants diagnosed with an intestinal injury or had radiographic findings of air in the intestinal wall, the portal vein, or in the abdomen, and those requiring abdominal drainage-laparotomy, were evaluated using hierarchical, unsupervised clustering analysis. Cases with congenital gastroschisis, omphalocele, intestinal atresia, and malrotation were excluded. Five major clusters of acquired neonatal intestinal injury were identified, each with unique features. Clearly, this approach opens new horizons in refining and classifying types of gut injury in neonates [94]. Unsupervised clustering applied in this manner provides the opportunity to interrogate each one of the clusters using integrated multiomics, which in turn generate systems biology networks from which comparative mechanisms can be determined and biomarkers for the individual clusters can be derived in a much more nuanced manner than if these are applied to all cases of intestinal injury currently termed NEC.

Several limitations should be taken into consideration regarding the application of AI in research on NEC. Any definition or ML model is only as good as the data and the definitions applied. In addition to the lack of an adequate and universally accepted definition for NEC, there are limitations regarding the use of AI in NEC research. These include the number of participating centers, issues with coding and subject identification, data analysis challenges (such as bias, retrospective nature, and missing values), and input variables (such as feeding mode, microbiome, type of surgery, as well as short- and long-term outcomes and quality of life). Lastly, NEC predictive models must be carefully defined according to their intended purpose and validated for reliability. Creating clinically significant models requires multidisciplinary collaboration.

Overall, the use of an ML algorithm combining routine clinical, laboratory, and gut tissue biomarkers could dramatically improve prediction/diagnosis and finally individualized management of neonates with intestinal injury. On the other hand, AI requires large, multicenter, and multimodal datasets of high quality for model training and testing. To this aim, large, multicenter studies, and mainly collaboration among international research centers, are needed so that a large amount of data is created (including the development of biobanks) that can be analyzed thereafter.

## 7. Clinical-Laboratory Difficulties and Diagnostic Dilemmas Related to NEC

In the context of NEC severity, early diagnosis and prompt treatment initiation is of utmost importance. Moreover, NEC should be distinguished from other gastrointestinal diseases that mimic NEC, such as SIP and food protein-induced enterocolitis syndrome, but have a different pathophysiology, prognosis, and management. However, the non-specific clinical presentation and laboratory findings make the diagnosis of NEC a challenging (or difficult) task in clinical practice [53,95,96]. Moreover, infection research in NEC cases indicates the involvement of systematic inflammatory response, while sepsis coexists in 20–30% of infants with NEC due to bacterial translocation secondary to disruption intestinal mucosal barrier [27]. For these reasons, the differential diagnosis of NEC from other inflammation-related intestinal diseases is difficult. To improve diagnostic ability, new biomarkers of inflammation in blood and urine, such as neutrophil CD64, or intestinal-specific proteins, such as the L-FABP, and a combination of them were studied [97,98]. A systematic review by Terrin et al. analyzed 22 studies that examined the diagnostic and prognostic ability of serum biomarkers of NEC. It was found that serum levels of certain proteins, predominantly proteins involved in inflammatory and immune responses, had high sensitivity and specificity either in the diagnosis of Bell stage II NEC or in the prediction of deterioration to advanced NEC. Still, none of the evaluated biomarkers had good accuracy in predicting the risk of NEC development or early diagnosing the disease, before the appearance of clinical signs. The authors dispute the early diagnostic and prognostic utility of the evaluated serum biomarkers due to the overall low quality of the analyzed studies [99].

These data show that, despite the ongoing research, important issues needing further clarification include the following: (a) which preterm infants are at high risk of developing NEC; (b) is early diagnosis before the development of clinical manifestations possible? (c) which is the prognosis of established advanced NEC, and (d) whether we can modify the outcome. For these reasons, novel accurate biomarkers capable of prediction, early diagnosis, differential diagnosis, and prognosis of NEC are urgently needed. 

The use of omics technology could help to detect molecules involved in disease pathogenicity and pathophysiology that could be utilized as reliable novel biomarkers with high positive and negative predictive values [35,98]. In addition, the omics could assist in the delineation of the mechanisms underlying NEC development [100,101] and potentially the pharmacotherapy [102] as well as the adverse outcomes of this severe neonatal disease. The multiple and complex mechanisms involved in inflammation and NEC/sepsis most probably require a multi-omics approach to better evaluate the systemic and tissue-specific responses [103]. 

## 8. OMICs

The term OMICs is an umbrella including a wide range of biological areas that investigate the various classes of biomolecules and the metabolic pathways linking the gene function with the subsequent metabolic stages, i.e., transcription and protein function, to the final metabolic products that define the phenotype, as well as the metabolic effects of the body microbiota and environmental factors. The main classes of omics include the metabolomics, proteomics, transcriptomics, genomics, microbiomics, and the microbiome metabolic products [103].

## 9. Metabolomics

Metabolomics is a bio-analytical approach for detecting and quantifying a large number of low molecular weight (<1000 Da) metabolites within biofluids, cells, and tissues, including substrates or products of metabolic pathways existing in all living organisms, such as amino acids, carbohydrates, and fatty acids. Because metabolites are the smallest and most specific components in the biological hierarchy, serving as downstream products of gene transcription and protein translation, they provide a more direct and detailed representation of the organism’s phenotype. The metabolome is the pattern of metabolites in a sample that represents the metabolic fingerprint, i.e., the phenotype of the underlying metabolic activity at a certain time point [35,104]. The analysis of metabolic profiles in human biological fluids and tissues allows us to instantly detect changes in the composition of a given set of endogenous conditions. 

Metabolomic analysis has certain advantages over other omics. Specifically, data collection does not require a priori defined metabolites under investigation. In general, there are two approaches for the detection of the metabolic profile: (a) non-targeted metabolomics is the hypothesis generating, global unbiased analysis of all small-molecule metabolites present within a biological system, under a given set of conditions. The non-targeted analysis is the identification of unknown metabolites, while there is no restriction in the number of metabolites that will be detected, and (b) the targeted analysis aims at a relatively small number of predefined metabolites. The non-targeted analysis reveals the metabolic fingerprint, i.e., a set of multiple biomarkers (without any restriction to certain molecules), which consists of a biologic pattern reflecting specific endo-phenotype or biopattern. In addition to understanding the pathophysiology (underlying biochemical processes) of several disease states, metabolomics may also contribute to early diagnosis and prognosis. Of note, the accurate identification of metabolites detected using metabolomics requires confirmation with known standards [105].

Metabolomic analysis can be performed in various biofluids, such as serum/plasma, urine, stool, cerebrospinal fluid, and intestinal tissue [53]. Perquisites for an accurate metabolomic analysis include high-quality specimens, availability of special equipment and skillfulness in advanced biotechnology, and adequate sample size. 

Metabolomics is conducted using various complementary analytical techniques. These include primarily mass spectrometry (MS) combined with gas or liquid chromatography (GC-MS, LC-MS), nuclear magnetic resonance (NMR) spectroscopy, as well as other advanced methods. The combination of these methods is necessary to comprehensively capture the metabolic profile as no single technique can detect all possible metabolites. Proton NMR (^1^H NMR) spectroscopy is particularly useful for analyzing small molecules and identifying unknown compounds [53,106,107]. 

Metabolite assessment involves several crucial steps: Initially, all detectable metabolites are identified using specific analytical techniques and then analyzed statistically. Following this, the chemical structures of the key metabolites are determined, and these metabolites are quantified and validated. Lastly, the data are interpreted by connecting the identified metabolites to the biological condition under investigation [53]. However, there are several limitations, such as the sample collection technique, the methodology and equipment used for analysis, and the challenges of handling the large volumes of data generated. Additionally, accurate analysis requires significant expertise. The metabolomic process is time-intensive, which limits its use for rapid diagnosis and treatment in clinical settings, and the initial cost of the necessary equipment is quite high.

## 10. The Role of Metabolomics in NEC Pathogenesis and Diagnosis

Despite advances in omics technologies over the last decade, the number of studies examining metabolomics in infants with NEC remains relatively small. Generally, these studies have focused on preterm infants with all stages of NEC or specifically stages II–III, utilizing different analytical platforms for untargeted and/or targeted metabolomic analyses. Other methodology issues include the small sample size and differences or an insufficient evaluation of NEC severity, as well as the presence of other co-morbidities, feeding modes, and drug administration. To avoid the invasiveness and risk of iatrogenic anemia associated with blood sampling in critically ill, especially very preterm infants, researchers have preferred using urine and feces specimens over blood. In some of these studies, the composition of the gut microbiota was also analyzed to help explain metabolomic findings potentially related to NEC. These investigations have been thoroughly reviewed elsewhere [53,108]. 

### 10.1. Metabolomic Studies in Infants NEC

Below is a summary of metabolomic studies conducted in infants with NEC, categorized by the type of biological fluid analyzed.

#### 10.1.1. Urine 

Very few studies have been conducted in NEC using urine metabolomics, alone. Thomaidou et al. using non-targeted ^1^H NMR spectroscopy and targeted LC-MS/MS analytical techniques, assessed the potential for the development of diagnostic biomarkers based on metabolomics alternation related to NEC. Results provided strong proof of evidence that the urine metabolome of neonates with NEC differs significantly at the clinical suspicion of the disease from that unaffected by the disease neonates. Twenty-five discriminant metabolites were identified as belonging to amino and organic acids, sugars, and vitamins, reflecting alternations in several metabolic pathways. Moreover, a set of metabolite combinations (tyrosine, arginine, riboflavin) was reported to have an excellent diagnostic performance in detecting neonates at risk for NEC [109].

The discriminative ability of longitudinal changes in urine metabolome was also evaluated by Picaud et al. in a prospective case-control study involving 6 VLBW infants with NEC and 12 matched controls, with or without feed intolerance. Using ^1^H NMR spectroscopy, they found that certain metabolites (lactate, betaine, myo-inositol, urea, creatinine, and N,N-dimethylglycine) could differentiate late-onset NEC from controls with good feed tolerance [110]. 

#### 10.1.2. Blood/Plasma/Serum 

Blood is expected to better reflect pathophysiological and metabolic changes in systematic illnesses, such as NEC and sepsis. Wilcock et al. conducted a non-targeted metabolomic study to identify potential biomarkers for early NEC diagnosis, involving 12 preterm infants (GA < 30 weeks) and 8 term controls. They analyzed two consecutive serum samples: one taken during the first week of life and another after the establishment of full feeds. The study found that 54 metabolites, primarily fatty acids and amino acids, showed significant differences between preterm and term infants. However, the metabolomic profile from the first week did not differ between preterm infants who later developed NEC and those who did not, indicating these early metabolites are not useful for predicting NEC. Conversely, the analysis of serum samples taken after full feeds revealed 16 metabolites that significantly differed between preterm infants who developed NEC and those who did not, as well as term controls. Seven of these increased metabolites (lysine, glycine, phenylalanine, decanoic acid, L-serine, methionine, and ornithine) were linked to IL-1beta upregulation [111]. 

A retrospective cohort study examined the association between early acylcarnitine patterns and NEC in seven preterm infants who developed the disease. Measurements were taken from dried blood specimens collected via heel-stick between 12 h and 8 days after birth. The study found that 14 acylcarnitine levels and acylcarnitine ratios were linked to an increased risk of developing NEC, suggesting that abnormal fatty acid metabolism is associated with both prematurity and the development of NEC. The authors concluded that newborn screening for these acylcarnitine patterns could serve as an objective biological marker for NEC risk, potentially aiding in disease prevention strategies [112].

Other studies evaluating the dried blood spot metabolome in preterm infants with NEC have shown that metabolic differences present at birth progress alongside significant variations in nutrition delivery, potentially contributing to the pathophysiology of NEC. Notably, a shift in the metabolite profile from predominantly amino acids at birth to acylcarnitines by day 42 was observed. Furthermore, day-one levels of alanine, phenylalanine, free carnitine, C16, arginine, C14:1/C16, and citrulline/phenylalanine were associated with the subsequent development of NEC [32].

In a recent study of serum metabolomics involving 17 neonates with NEC (all stages) and 15 LOS, L-carnitine was found to efficiently discriminate NEC cases from controls. The study noted that neonates with NEC had increased L-carnitine levels, likely due to the increased cellular demand for energy production from fatty acids and as a compensatory mechanism to mitigate the toxic effects of free oxygen radicals. In contrast, blood levels of phosphatidylcholines and lysophosphatidylcholines were significantly reduced in neonates with NEC compared to controls. Interestingly, since certain bacteria can synthesize phosphatidylcholines, the authors could not rule out a bacterial origin for these substances in neonates with NEC [101]. 

In general, alterations in fatty acid metabolism (including acyl-carnitines involved in their transport into the mitochondria for beta-oxidation) and amino acids are considered key findings in metabolic studies conducted on infants with NEC [108]. 

Stewart et al. combined serum metabolomics and proteomics to investigate potential differences in protein patterns and RNA expression between infants with NEC (n = 6), LOS (n = 4) and controls (n = 10). The study found no significant differences in serum metabolomic and proteomic profiles between infants with NEC and controls. Additionally, no metabolites or proteins were exclusively present in neonates with NEC or LOS that were absent in controls. The variation in protein expression among diseased infants, and the individuality in response to NEC/LOS, may be due to differing disease pathophysiology, highlighting the challenges in classifying patients into specific disease types [113,114]. 

#### 10.1.3. Feces

Scientific interest has also switched to fecal metabolomics in NEC to gain insights into the disease’s pathophysiology and identify potential biomarkers.

In this context, Rusconi et al. conducted a metabolomics study on feces obtained 1–5 days before NEC developed in nine neonates with NEC stages II–III and 19 GA- and BW-matched controls. They identified 764 metabolites involved in six pathways that differed between cases and controls. The study focused on sphingolipid metabolism due to its relationship with inflammatory disorders. It was found that neonates with NEC had decreased ceramides and increased sphingomyelins compared to controls. However, the identified metabolites provided only 73% accuracy, as hierarchical clustering of sphingolipids included only 60% of the NEC cases. Despite the detected differences in sphingolipids in pre-NEC stool between cases and controls, further verification of the data through larger studies is needed [115].

In another multicenter prospective case-control study, fecal samples from preterm infants (GA < 30 weeks) were longitudinally collected after birth. Samples taken 1–3 days before the clinical manifestations of severe NEC (Bell’s stage III) were analyzed in 31 cases and an equal number of GA- and postnatal age-matched controls using targeted high-performance liquid chromatography. Significant fecal alterations were observed in NEC cases compared to controls, including increased levels of five essential amino acids (isoleucine, leucine, methionine, phenylalanine, and valine), while lysine and ethanolamine ratios were lower. A set of five amino acids moderately predicted NEC. However, the relationship between these changes and the development of NEC, and the potential role of amino acids as NEC biomarkers, remains to be elucidated [116].

#### 10.1.4. Fecal Volatile Organic Compounds

Other researchers have evaluated the potential of changes in fecal volatile organic compounds (VOCs) as biomarkers for NEC using untargeted GC-MS or electronic nose techniques. In a multicenter prospective study conducted across eight neonatal units in the United Kingdom, Probert et al. analyzed daily fecal samples for VOCs in preterm infants (<34 weeks GA), of whom 49 subsequently developed definite NEC. The results from 32 NEC cases were compared with samples from controls without NEC. The study found that three of the nine VOC cluster groups were associated with NEC and could predict disease development up to 3–4 days before clinical diagnosis. Further discriminant analyses identified five individual VOCs that could predict NEC risk up to 4 days before clinical diagnosis, with an area under the receiver operating characteristic (AUC) curve of 0.75–0.76 [117]. Additional studies examining VOCs reported similar results [118,119]. 

### 10.2. Studies Combing Metabolomics and Gut Microbiome

In the first (2013) prospective study on urine metabolomics (NMR) in preterm infants with NEC (defined as modified Bell’s stages II–III), Morrow et al. also profiled stool bacterial communities using 16S rRNA gene sequencing. This study involved 11 infants under 29 weeks gestational age who developed NEC and 21 matched survivors without NEC (control group). Significant differences in microbial diversity and microbiome composition were observed between the NEC group and the controls, suggesting that early dysbiosis plays a crucial role in the pathobiology of NEC. Interestingly, metabolomic analysis of urine samples obtained on days 4 to 9 revealed no single metabolite present in all NEC cases. However, a high urinary alanine to histidine ratio was associated with microbial dysbiosis and was a good predictor of overall NEC occurrence [120].

Cases of NEC stage I are often excluded from studies due to uncertainty about whether infants have an underlying NEC pathophysiology. Brehin et al. conducted a prospective study on the gut microbiome and metabolome in 22 preterm infants focusing on suspected NEC (stage I NEC). The results showed that NEC stage I is characterized by a distinct gut microbiota, microbiome, and gut microbial metabolite profile. In infants presenting symptoms within the first 10 days, the gut microbiome had already started to diverge, appearing more homogeneous compared to healthy infants, who had a higher abundance of Klebsiella species. Although no significant changes were observed in fecal metabolites during the specific period, this early divergence can be a critical period for the progression of gut injury, possibly reversible by clinical measures (e.g., increasing the enteral volume of nutrition). In the second 10 days of life, infants with NEC showed significantly lower levels of serine in feces and a higher abundance of Streptococcus species and bacteria from the Micrococcales order. During the third 10-day postnatal period, changes were more pronounced in NEC cases, with increased levels of Staphylococcus and Streptococcus species. By the second month of life, taxonomical differences in the gut microbiota included an increase in Raoultella species in NEC cases, which also exhibited significantly lower fecal levels of ethanol and leucine [121].

Du et al. investigated the discriminative potential of the gut microbiome and microbiome-derived tricarboxylic acid metabolites to distinguish NEC from other gastrointestinal diseases. Fecal samples were collected at enrollment from preterm infants (GA ≤ 34 weeks); 16 with NEC (stage II or above) and 16 with non-NEC abdominal manifestations. The study found a significant decrease in unclassified Staphylococcus, Lactobacillaceae, and Bifidobacterium animalis subsp. lactis in the NEC group (*p* < 0.05). Metabolomic analysis revealed significantly increased levels of three tricarboxylic acid metabolites (succinate, L-malic acid, and oxaloacetate) in infants with NEC, with areas under the ROC curve of 0.6641, 0.7617, and 0.7344, respectively. These findings suggest that specific microbial patterns and certain Krebs cycle metabolites may play a role in the early diagnosis of NEC [122]. It should be noted, however, that tricarboxylic acid metabolites are not only products from human cellular metabolism but also intermediates from bacterial fermentation [123].

Similarly, Xiong et al. evaluated whether differences in gut microbiota composition and short-chain fatty acids (SCFAs) could distinguish between NEC and food protein-induced allergic proctocolitis. The study included 43 infants, 22 with NEC and 21 with allergic proctocolitis. The bacterial composition and levels of certain fatty acids in stool differed significantly between the groups. Specifically, Halomonas, Acinetobacter, Bifidobacterium, and Stenotrophomonas were more prevalent in the NEC group. NEC infants had significantly lower levels of acetic acid, propionic acid, butyric acid, isovaleric acid, and total SCFAs but higher levels of hexanoic acid compared to the allergic proctocolitis group [124]. SCFAs derived from the fermentation of dietary fibers (e.g., acetic acid, butyrate, and propionic acid) were found to have significant immune properties promoting gut homeostasis after binding to the ‘metabolite-sensing’ receptors [125].

Interestingly, other investigators found no consistent association between the intestinal microbiome and the risk of sepsis or NEC. They observed significant changes in bacterial composition over the first 6 weeks of life. Multiple regression analysis revealed that individuality was the strongest factor associated with intestinal colonization, followed by delivery mode. Other factors, including health status (presence of infection or NEC), length of NICU stay, and feeding mode, were not significantly associated with bacterial composition. The authors concluded that the microbiome and metabolome of preterm infants are personalized and mainly consist of species prevalent in antibiotic-treated infants, rather than being linked to disease state [126]. 

In a recent systematic review by Moschino et al. on the metabolome and gut microbiota for predicting NEC and SIP, 21 out of 27 studies found significant differences in microbiome composition between NEC and non-NEC infants, while six studies did not demonstrate any significant changes in fecal microbiome patterns between the two groups [35]. 

## 11. Limitations

There are several limitations that the authors of this narrative review must acknowledge. Firstly, the potential for bias may exist due to the selection of articles and the authors’ interpretation. Unlike a systematic review, we did not follow a structured methodology for literature selection, which could result in incomplete or selective inclusion of studies, with some important articles potentially overlooked. Lastly, the broad scope of the information presented may have limited the depth of detail on certain key issues, especially when studies with significant heterogeneity are included, as in our case.

## 12. Conclusions

Many neonatal gut injuries, classed under the umbrella diagnosis of NEC, share clinical symptoms, making it difficult to distinguish between them based on clinical presentation alone. NEC is a complex and multifactorial diagnosis with varying presentations and underlying mechanisms, complicating efforts to classify it into distinct subtypes. The absence of reliable, specific biomarkers for different forms of neonatal gut injury hampers accurate subtyping and early diagnosis. 

**Figure 1:**
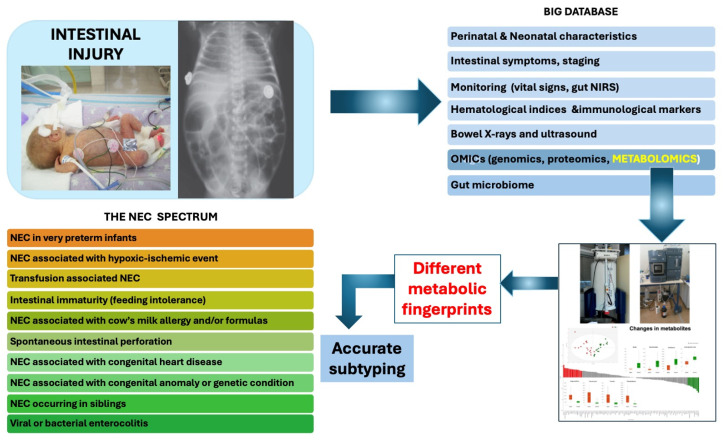
A conceptual model for applying metabolomics to clarify different types of neonatal intestinal injury within the NEC spectrum, enabling precise categorization of each condition.

In response to the theoretical question of whether metabolomics could enhance our understanding of the NEC spectrum, it is our opinion that this approach holds significant promise (Figure 1). By analyzing the distinct metabolic profiles associated with NEC, metabolomics provides valuable insights into the biochemical changes occurring in affected infants. Metabolomics could help identify specific biomarkers that differentiate various forms of intestinal injury, potentially enabling earlier and more accurate diagnoses. Additionally, metabolomics offers a deeper understanding of the disease’s underlying mechanisms, which could inform the development of more targeted and effective treatments. Integrating metabolomics with microbiota and other omic analyses should provide a comprehensive understanding of these various forms of intestinal injury by linking metabolic changes to specific microbial communities and host responses.

While several metabolites have been associated with NEC, there remains considerable variability among studies, and no single metabolite or combination has consistently demonstrated reliable diagnostic or prognostic value as a biomarker for NEC. To achieve definitive conclusions, larger, well-designed studies involving sufficient numbers of infants with NEC and matched controls are essential. Furthermore, integrating metabolomics with clinical, microbiological, metabolomic, and genetic data could provide a more comprehensive understanding of NEC and help identify robust biomarkers for early diagnosis and intervention. In any case, the accurate subtyping of the different forms of neonatal gut injury is essential if we are to make significant progress in understanding the complex condition known as “NEC”, and this remains an ongoing challenge.

## Data Availability

Not applicable.
